# A Case of COVID-19 Infection With Delayed Thromboembolic Complication on Warfarin

**DOI:** 10.7759/cureus.8847

**Published:** 2020-06-26

**Authors:** Alpana Garg, Sachin Goyal, Pragnesh Patel

**Affiliations:** 1 Internal Medicine, Wayne State University Detroit Medical Center, Detroit, USA; 2 Gastroenterology, Wayne State University, Detroit, USA; 3 Internal Medicine, Wayne State University, Detorit, USA

**Keywords:** covid 19, pulmonary embolism (pe), systemic anticoagulation, discharge, heparin, warfarin

## Abstract

Novel coronavirus disease 2019 (COVID-19) pandemic has posed an unprecedented threat to humanity with more than eight million infections and 450,000 deaths reported worldwide so far. The spectrum of the disease varies from mild asymptomatic infection to severe disease with rapid progression to acute respiratory distress syndrome and multiorgan failure. It is associated with a prothrombotic state and hence there is a risk of thromboembolic complications in critically ill patients, even after recovery. However, the duration of prothrombotic risk after recovery is yet to be determined. We present the case of a 78-year-old man with a history of atrial fibrillation on warfarin who had been recently discharged to a nursing home after recovering from COVID-19 pneumonia and presented to the emergency department a month later with worsening shortness of breath and cough. He was found to have worsening respiratory failure with multiple segmental pulmonary emboli, despite being on warfarin, and supratherapeutic international normalized ratio (INR). He required mechanical ventilation and was started on steroids and therapeutic enoxaparin anticoagulation. This case highlights the risk of delayed thromboembolic complications in patients with COVID-19 infection and the need to identify the subgroup of patients with a higher risk of thromboembolism, such as discharges to nursing homes and those in need of oxygen requirement; and those with underlying comorbid conditions that may require anticoagulation for a longer duration. The role of heparin is being increasingly investigated in patients with COVID-19 infection; however, the role of other anticoagulants such as warfarin is yet to be defined.

## Introduction

Coronavirus disease 2019 (COVID-19) is rapidly spreading across the globe. It was declared a pandemic by the World Health Organization on March 11, 2020, and within a few months, more than eight million cases and 450,000 deaths have been reported worldwide [1,2]. COVID-19 primarily targets the lungs, and the clinical spectrum varies from mild disease to severe disease including acute viral pneumonia, acute respiratory distress syndrome, septic shock, multiorgan failure, and even death [3]. However, our current understanding of its pathophysiology and complications is limited and rapidly evolving. There is evidence to suggest that COVID-19 is associated with a prothrombotic state with increased risk of venous and arterial thrombi including pulmonary embolism, deep venous thrombosis, and systemic arterial embolisms [4]. The role of therapeutic anticoagulation during the hospitalization for COVID-19 is increasingly being investigated [5,6]. Some of the earliest studies have shown improved outcomes with therapeutic anticoagulation in this group of patients [7,8]. We present a unique case of COVID-19 infection with delayed thromboembolic complication.

## Case presentation

Our patient, a 78-year-old man, came from a nursing home to the emergency department with progressively worsening shortness of breath and nonproductive cough of two days' duration. He denied any fever, chills, nasal discharge, chest pain, or any abdominal complaints. He had been admitted to a hospital one month back (during the peak of COVID-19 pandemic in Detroit) with a runny nose, low-grade fever, and shortness of breath. At that time, he had tested positive for COVID-19, had been given a hydroxychloroquine course (for five days), and discharged to the nursing home, with stable oxygen requirements, on 4 L of oxygen through the nasal cannula. He had not required any mechanical ventilation or intensive care monitoring during hospitalization.

His past medical history included hypertension, diabetes, obesity (body mass index of 35), atrial fibrillation (with a CHA_2_DS_2_-VASc score of 4), renal failure secondary to diabetic nephropathy post-renal transplant (15 years back), and interstitial lung disease (ILD). The etiology of fibrotic changes in the lung was thought to be drug-induced (sirolimus); however, the changes had been stable over the last three years and he had been off the drug for the past few years. There had been no history of any thrombotic events including deep vein thrombosis or pulmonary embolism in the past. He denied any use of alcohol, tobacco, or any illicit drugs. His medications included insulin, tacrolimus, warfarin, and multivitamins. He denied any recent dose adjustment of warfarin. All his medications had been continued and no changes in anticoagulation had been made during the recent hospital discharge.

**Figure 1:**
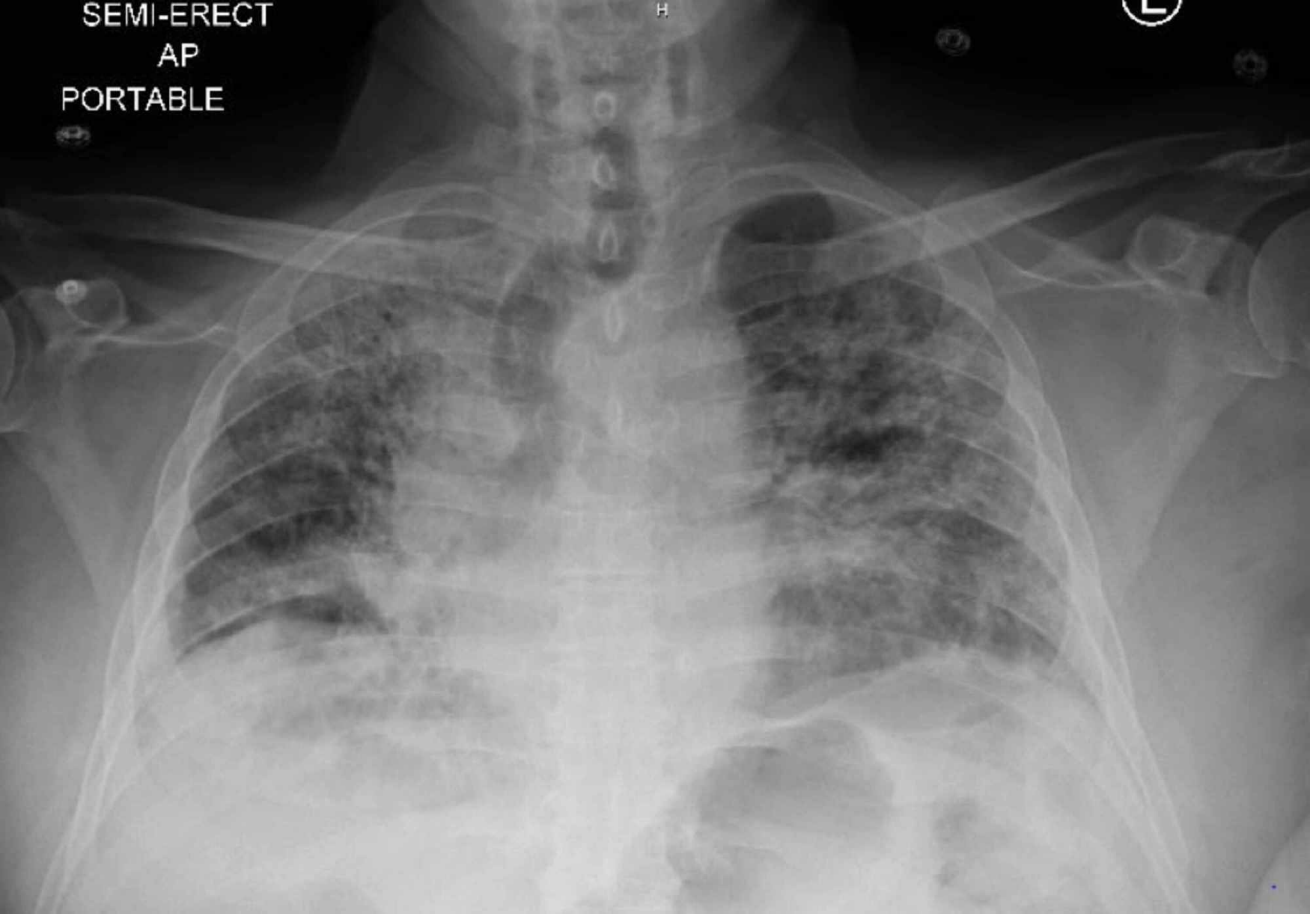
Chest X-ray showing bilateral interstitial infiltrates

During the current presentation, he was afebrile with a heart rate of 120/min, blood pressure of 120/80 mmHg, respiratory rate of 24/min, and 90% oxygen saturation on 4 L of the nasal cannula. He had coarse breath sounds bilaterally, was using accessory muscles of respiration, and his pulse was irregular with tachycardia. The patient was immediately tested for COVID-19 with a nasopharyngeal swab, which was negative. However, based on the current clinical presentation, the possibility of a false-negative test result, and his recent COVID-19 infection, he was immediately placed on contact and airborne precautions for possible COVID-19 pneumonia. His chest X-ray showed bilateral opacities suggestive of multifocal pneumonia (Figure 1)

Electrocardiogram indicated atrial fibrillation without rapid ventricular response. He was given metered-dose inhaler breathing treatments, supplemental oxygen through the nasal cannula, and was started on vancomycin and cefepime. Blood cultures and other lab work were obtained (Table 1). He was found to have mild normocytic normochromic anemia, prolonged prothrombin time, activated plasma thrombin time, elevated international normalized ratio (INR), and hyperglycemia. The inflammatory markers including C-reactive protein (CRP), ferritin, and D-dimer were elevated with mild high sensitivity troponin elevation.

**Table 1:** Laboratory results during current admission

Investigation	Value	Investigation	Value
Sodium (mMol/L; ref range: 136-145)	134	Hemoglobin (gm/dL; ref range: 13.3-17.1)	11.9
Potassium (mMol/L; ref range: 3.5-5.1)	4.9	Platelets (K/CUMM; ref range: 150-450)	227
Chloride (mMol/L; ref range: 98-107)	102	Mean corpuscular volume (FL; ref range: 81-98)	92.3
Bicarbonate (mMol/L; ref range: 21-31)	24	Mean corpuscular hemoglobin (pg; ref range: 27.1-34.0)	29.5
Glucose (mg/dL; ref range: 75-105)	337	Mean corpuscular hemoglobin concentration (%; ref range: 32.6-35.4)	31.9
Blood urea nitrogen (mg/dL; ref range: 7-25)	33	White blood cells (K/CUMM; ref range: 3.5-10.6)	9.8
Creatinine (mg/dL; ref range: 0.7-1.30)	1.1	Prothrombin time (seconds’ range: 9.4-11.7)	38.5
Magnesium (mg/dL; ref range: 1.6-3.0)	1.7	International normalized ratio	6.30 (improved to 3 after 24 hours)
Phosphorus (mg/dL; ref range: 2.5-4.5)	3.8	Activated plasma thrombin time (seconds’ range: 23.1-33.1)	36.3
Aspartate aminotransferase (Units/L; ref range: 13-39)	22		
Alanine aminotransferase (Units/L; ref range: 7-52)	26		
Alkaline phosphatase (Units/L; ref range: 45-115)	99		

A comparison of inflammatory markers during current and previous admission is shown in Table 2.

**Table 2:** A comparison of inflammatory markers for COVID-19 test between previous admission and current hospitalization RT-PCR: reverse transcription-polymerase chain reaction

Inflammatory markers for COVID-19	Previous admission (30 days back)	Current admission
COVID-19 nasopharyngeal RT-PCR	Positive	Negative
C-reactive protein (mg/L; normal high: <5.0)	107.9	92.9
Lactate dehydrogenase (Units/L; ref range: 140-271)	309	252
D-diimer (mg/L; normal high: <0.50)	1.49	1.32
Ferritin (ng/mL; ref range: 23.9-336.2)	819.4	460.2
White blood cells (K/CUMM; ref range: 3.5-10.6)	2.6	9.8
Absolute lymphocyte count (K/CUMM; ref range: 1.0-3.8)	0.8	1.4
High sensitivity troponin (ng/L; ref range: 3-17)	16	22

The patient was started on high dose of methylprednisolone intravenously. Since his INR was supratherapeutic, warfarin was held. However, within the next 24 hours, the respiratory status of the patient worsened, necessitating higher oxygen requirements. An arterial blood gas analysis showed pH 7.402/PCO2 40.2/PO2 70.2 and oxygen saturation of 90% at 6 L of nasal cannula. A decision was made to start the patient on high-flow nasal cannula and transfer him to intensive care unit. In the next two hours, his repeat arterial blood gas analysis showed pH 7.31/PCO2 55.7/PO2 60.7, which was suggestive of hypoxemia and carbon dioxide retention, and a decision for tracheal intubation and mechanical ventilation was taken.

Since the patient was on tacrolimus, there was also concern for opportunistic infections or superimposed bacterial pneumonia; however, the infectious workup including respiratory culture, viral panel, blood cultures, fungal cultures, streptococcal antigen, and pneumocystis stain of sputum came back negative. Although clinical suspicion for COVID-19 pneumonia was high given his history, clinical presentation, and imaging, repeat testing of nasopharyngeal secretions for COVID-19 was still negative. False-negative results of RT-PCR for severe acute respiratory syndrome coronavirus 2 (SARS-CoV-2) have been reported in studies [9,10]. No additional experimental therapy (including remdesivir, tocilizumab) was administered for COVID-19 pneumonia as he did not meet the criteria for starting treatment based on the hospital protocol followed at the time.

**Figure 2:**
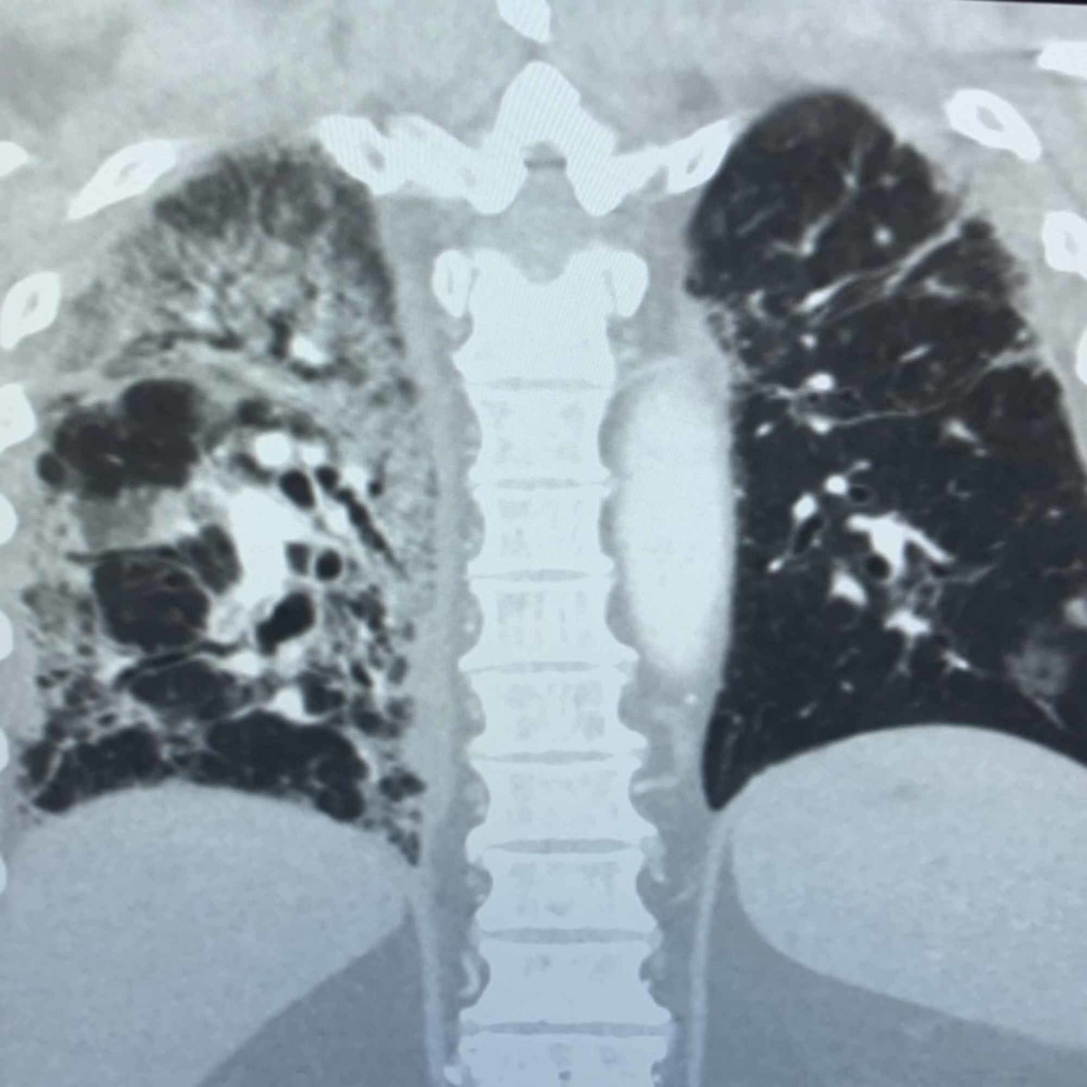
Coronal chest CT showing ground-glass opacities superimposed on diffuse fibrotic changes of the lung parenchyma with traction bronchiectasis in both upper and lower lobes CT: computed tomography

**Figure 3:**
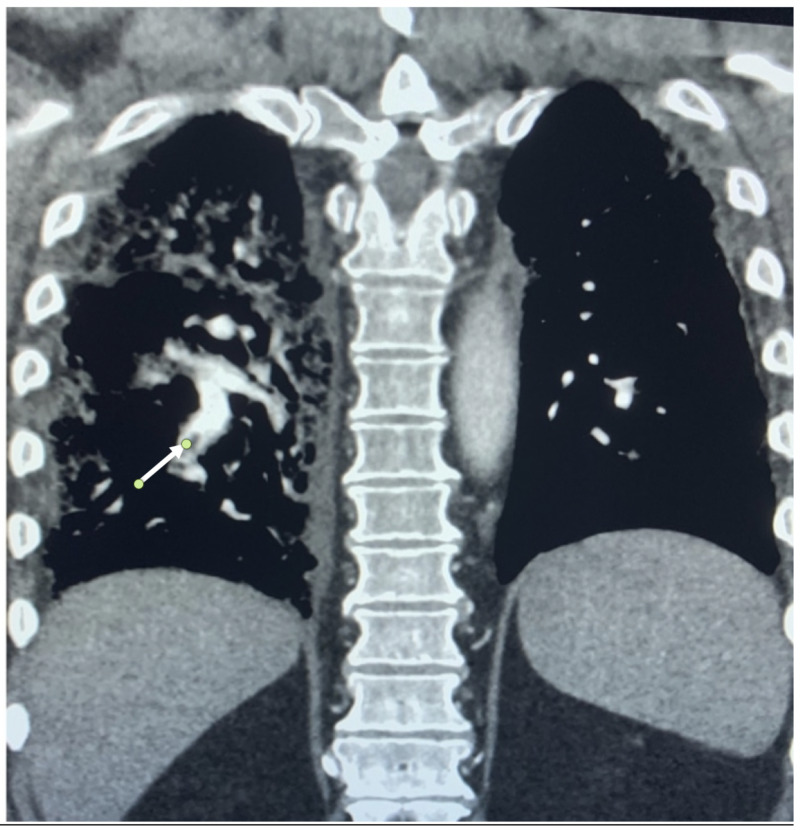
Coronal chest CT showing filling defect (white arrow) within the right lower lobe segmental pulmonary artery CT: computed tomography

Lung imaging with a CT scan was obtained (Figure 2), which showed multifocal ground-glass opacities concerning for multifocal pneumonia superimposed on previous fibrotic changes. In addition, there were multiple segmental pulmonary emboli on the right side of the lung (Figure 3).

Two-dimensional echo did not show any evidence of right heart strain, regional wall motion abnormalities, or thrombus. The estimated ejection fraction was 55-60%. At that time, a repeat INR was also obtained, which was found to be 3, and the patient was started on enoxaparin therapeutic anticoagulation dosing (1 mg/kg twice daily). Lower extremity Doppler was negative for deep vein thrombosis. Thrombophilia workup was not done given the acute onset of the embolus and was planned for later in the outpatient setting.

Over the next few days, his ventilator requirements decreased, and he did well with a spontaneous breathing trial and was subsequently extubated after a total intubation duration of five days. His oxygen requirement also decreased over the next few days although he continued to require oxygen through a nasal cannula. He was subsequently transferred to the medical floor and then discharged a week later. Given he had supratherapeutic INR on presentation with unclear etiology, new multiple pulmonary emboli while on warfarin, and based on recent guidelines supporting the use of direct oral anticoagulants (DOACs) in atrial fibrillation, it was decided that he will be started on apixaban on discharge [11].

## Discussion

This was a rare case of COVID-19 with atrial fibrillation, and our patient was already on anticoagulation and presented with the delayed complication of pulmonary embolism. He had previously tested positive for COVID-19 and presented a month later with worsening hypoxia and respiratory failure requiring mechanical ventilation. The etiology was indeed multifactorial with underlying lung disease, superimposed recent viral infection (as suggested by imaging with lung infiltrates), and new multiple pulmonary emboli. It is interesting to note that COVID-19 testing repeated twice came back negative, but it is quite possible that COVID-19 infection had never resolved.

The present case highlights the importance of recognizing pulmonary embolism as a later complication in patients who were recently treated for COVID-19 pneumonia and discharged from the hospital. Based on the emerging literature on thromboembolic complications associated with the infection, patients who are critically ill with COVID-19 pneumonia are at risk of developing pulmonary embolism [12]. The development of new pulmonary embolism in such patients who are already compromised with pre-existing respiratory conditions can further contribute to existing respiratory failure with an increase in morbidity and mortality.

COVID-19 also may predispose people to venous thromboembolism by several mechanisms, including endothelial dysfunction, which is characterized by increased levels of von Willebrand factor, systemic inflammation by Toll-like receptor activation, and a procoagulant state by tissue factor activation [13]. The role of heparin is especially being investigated as it may exert anti-viral properties by binding to the viral proteins and inhibiting viral attachment [14]. Current data support the use of low-molecular-weight heparin in these patients. D-dimer levels may be a useful marker of severity of disease in COVID-19 patients; however, to date, there is no consensus on how it should be used for the management and/or monitoring of COVID-19 patients or with respect to anticoagulation [15]. Full-dose anticoagulation in patients admitted with COVID-19 has shown survival benefits in a recent study including 2,773 patients. Moreover, a longer duration of treatment with anticoagulants was associated with a reduced risk of mortality [7]. However, the duration of full-dose anticoagulation needs further elaboration to prevent delayed thrombi formation, as seen in our patient. Hospitalization for medical illness increases the risk of venous thromboembolic events by several folds and post-discharge thromboprophylaxis is not fully utilized [16]. It is important to identify the subgroup of patients who are at risk of thromboembolic complications and need anticoagulation after discharge from the hospital setting. Our patient had multiple preexisting comorbid conditions, with his recent hospitalization and nursing home stay, and new respiratory failure after COVID-19, which increased the risk of developing pulmonary embolism. He developed thromboembolism, despite being on warfarin, and supratherapeutic INR. Larger prospective studies are needed to confirm the choice and duration of anticoagulation in patients after recovery from COVID-19.

## Conclusions

It is important to timely diagnose pulmonary embolism and other thromboembolic complications in patients with COVID-19. The duration of the prothrombotic state with COVID-19 is not yet known, and hence thromboprophylaxis in all patients and therapeutic anticoagulation based on risk assessment during the hospital stay or at the time of discharge should be considered on a case-by-case basis. There is a need to identify patients recovering from COVID-19 who are at risk of thromboembolism and will benefit from a longer duration of therapeutic anticoagulation after recovery from critical illness. Further studies are needed to understand the role of heparin, enoxaparin, warfarin, and DOACs for prophylaxis and therapeutic anticoagulation in patients with COVID-19.

## References

[1] Cucinotta D, Vanelli M (2020). WHO declares COVID-19 a pandemic. Acta Biomed.

[2] (2020). COVID-19 Dashboard by the Center for Systems Science and Engineering (CSSE) at Johns Hopkins University (JHU). https://coronavirus.jhu.edu/map.html.

[3] Wu Z, McGoogan JM (2020). Characteristics of and important lessons from the coronavirus disease 2019 (COVID-19) outbreak in China: summary of a report of 72314 cases from the Chinese Center for Disease Control and Prevention (Epub ahead of print). JAMA.

[4] Klok FA, Kruip MJHA, van der Meer NJM (2020). Confirmation of the high cumulative incidence of thrombotic complications in critically ill ICU patients with COVID-19: an updated analysis. Thromb Res.

[5] Kollias A, Kyriakoulis KG, Dimakakos E, Poulakou G, Stergiou GS, Syrigos K (2020). Thromboembolic risk and anticoagulant therapy in COVID-19 patients: emerging evidence and call for action. Br J Haematol.

[6] Connors JM, Levy JH (2020). COVID-19 and its implications for thrombosis and anticoagulation. Blood.

[7] Paranjpe I, Fuster V, Lala A (2020). Association of treatment dose anticoagulation with in-hospital survival among hospitalized patients with COVID-19 (Epub ahead of print). J Am Coll Cardiol.

[8] Tang N, Bai H, Chen X, Gong J, Li D, Sun Z (2020). Anticoagulant treatment is associated with decreased mortality in severe coronavirus disease 2019 patients with coagulopathy. J Thromb Haemost.

[9] Xiao AT, Tong YX, Zhang S (2020). False-negative of RT-PCR and prolonged nucleic acid conversion in COVID-19: rather than recurrence (Epub ahead of print). J Med Virol.

[10] Yan Y, Chang L, Wang L (2020). Laboratory testing of SARS-CoV, MERS-CoV, and SARS-CoV-2 (2019-nCoV): current status, challenges, and countermeasures. Rev Med Virol.

[11] January CT, Wann LS, Calkins H (2019). 2019 AHA/ACC/HRS Focused Update of the 2014 AHA/ACC/HRS guideline for the management of patients with atrial fibrillation: a report of the American College of Cardiology/American Heart Association Task Force on Clinical Practice Guidelines and the Heart Rhythm Society. Heart Rhythm.

[12] Wichmann D, Sperhake JP, Lütgehetmann M (2020). Autopsy findings and venous thromboembolism in patients with COVID-19 (Epub ahead of print). Ann Intern Med.

[13] Giannis D, Ziogas IA, Gianni P (2020). Coagulation disorders in coronavirus infected patients: COVID-19, SARS-CoV-1, MERS-CoV and lessons from the past. J Clin Virol.

[14] Alijotas-Reig J, Esteve-Valverde E, Belizna C (2020). Immunomodulatory therapy for the management of severe COVID-19. Beyond the anti-viral therapy: a comprehensive review. Autoimmun Rev.

[15] (2020). American Society of Hematology: COVID-19 and D-dimer: frequently asked questions. https://www.hematology.org/covid-19/covid-19-and-d-dimer.

[16] Mahan CE, Fisher MD, Mills RM, Fields LE, Stephenson JJ, Fu AC, Spyropoulos AC (2013). Thromboprophylaxis patterns, risk factors, and outcomes of care in the medically ill patient population. Thromb Res.

